# Does Bisphenol A Confer Risk of Neurodevelopmental Disorders? What We Have Learned from Developmental Neurotoxicity Studies in Animal Models

**DOI:** 10.3390/ijms23052894

**Published:** 2022-03-07

**Authors:** Chloe Welch, Kimberly Mulligan

**Affiliations:** 1 Division of Biological Sciences, University of California, San Diego, 9500 Gilman Drive, La Jolla, CA 92093, USA; cwelch@ucsd.edu; 2 Department of Biological Sciences, California State University, Sacramento, 6000 J Street, Sacramento, CA 95819, USA

**Keywords:** bisphenol A, endocrine disruptors, neurodevelopmental disorder, neural stem cell development, synaptogenesis, synaptic plasticity, behavior

## Abstract

Substantial evidence indicates that bisphenol A (BPA), a ubiquitous environmental chemical used in the synthesis of polycarbonate plastics and epoxy resins, can impair brain development. Clinical and epidemiological studies exploring potential connections between BPA and neurodevelopmental disorders in humans have repeatedly identified correlations between early BPA exposure and developmental disorders, such as attention deficit/hyperactivity disorder and autism spectrum disorder. Investigations using invertebrate and vertebrate animal models have revealed that developmental exposure to BPA can impair multiple aspects of neuronal development, including neural stem cell proliferation and differentiation, synapse formation, and synaptic plasticity—neuronal phenotypes that are thought to underpin the fundamental changes in behavior-associated neurodevelopmental disorders. Consistent with neuronal phenotypes caused by BPA, behavioral analyses of BPA-treated animals have shown significant impacts on behavioral endophenotypes related to neurodevelopmental disorders, including altered locomotor activity, learning and memory deficits, and anxiety-like behavior. To contextualize the correlations between BPA and neurodevelopmental disorders in humans, this review summarizes the current literature on the developmental neurotoxicity of BPA in laboratory animals with an emphasis on neuronal phenotypes, molecular mechanisms, and behavioral outcomes. The collective works described here predominantly support the notion that gestational exposure to BPA should be regarded as a risk factor for neurodevelopmental disorders.

## 1. Introduction

Bisphenol A (BPA, 2,2-bis (4′-hydroxyphenyl) propane), a ubiquitous chemical used in the synthesis of polycarbonate plastic and epoxy resins, is taking shape as a risk factor for neurodevelopmental disorders (NDDs). NDDs refer to a heterogeneous group of nervous system disorders caused by changes in brain development, including autism spectrum disorder (ASD), attention deficit/hyperactivity disorder (ADHD), intellectual disability (ID), learning disabilities, cerebral palsy, seizure disorders, and impairments in vision and hearing. Analysis of the prevalence of developmental disabilities in the United States (U.S.) between 2009 and 2017 indicated that 16.93% of children were diagnosed with an NDD [1]. Studies have also found that the prevalence of NDDs has increased significantly over the past several decades—most notably, the incidence ASD has increased by almost 300 percent in the last 20 years [2,3].

NDDs have complex etiologies and can result from both genetic susceptibilities and environmental factors [4,5]. Given the increasing prevalence of BPA in our environment [6], along with numerous studies showing an association between human BPA exposure and NDDs [7,8,9], BPA has garnered increasing attention over the past fifteen years for its ability to disrupt brain development. The aim of this review is to summarize studies that have delineated neurodevelopmental consequences of early (prenatal or perinatal) BPA exposure in animal models in an effort to illuminate the mechanisms by which it may impede human brain development. 

BPA is categorized as an endocrine disrupting chemical (EDC) due to its ability to bind endogenous hormone receptors and cause adverse effects. Structurally similar to estradiol, BPA is most well-known for its ability to agonize and antagonize estrogen receptor (ER) subtypes and antagonize androgen receptors [10,11]. BPA also elicits non-estrogenic/non-androgenic impacts on development by binding other receptors, including the thyroid hormone receptor [12,13], glucocorticoid receptor [14,15,16], the G protein-coupled receptor, GPR30 [17] and Peroxisome Proliferator-Activated Receptor γ (PPARγ) [18,19]. The ability of BPA to influence hormonal signaling was first documented in the 1930s [20,21]. Despite awareness of its endocrine disrupting capability, BPA was adapted for use in the synthesis of polycarbonate plastics in the 1950s and quickly became one of the most prevalent synthetic compounds in the world [6,22]. An estimated 7.7 million metric tons of BPA were generated worldwide in 2015, and production is expected to rise to 10.6 million metric tons in 2022 [6]. 

BPA enters the body via ingestion, dermal absorption, and inhalation, with ingestion being the most common route of exposure [23,24,25]. A wide array of products used in everyday life contain BPA, including plastic containers, thermal papers, food cans, and beverage cans [26,27,28]. Residual BPA can leach from these products due to incomplete polymerization during production or from depolymerization when exposed to high temperatures or extreme pH conditions, which speed up the hydrolysis of ester bonds that link BPA monomers [6]. 

Due to concerns surrounding the endocrine disrupting capabilities of BPA, the European Union banned its use in all infant products beginning in 2011, and the U.S. Food and Drug Administration followed suit in 2012. However, BPA remains pervasive in our environments; of greatest concern, pregnant women are still persistently exposed to BPA in a variety of ways. The lipophilic structure of BPA allows it to readily cross cell membranes, as well as both placental and blood–brain barriers [29,30,31], enabling its ability to potentially affect the neurodevelopmental program of a growing embryo or fetus.

Despite vast evidence that BPA should be more tightly regulated, establishing safe exposure levels is complicated as is evidenced by the varied reference doses in different countries. For example, the current reference dose set by the Environmental Protection Agency in the U.S. is 50 mg/kg/day [32], the set tolerable dose intake (TDI) in Canada is 25 mg/kg/day [33], while the European Food Safety Authority (EFSA) set their TDI at 4 mg/kg/day [34]. 

The reference doses of BPA may be reasonable limits for adults, but the research summarized in this review and others suggest that doses in the ng/kg/day range may have deleterious impacts on the developing brains of laboratory animals [35]. In adult humans, BPA does not bioaccumulate and is metabolized and excreted within 48 h; however, given its environmental prevalence, many individuals experience chronic exposure. In addition, while BPA exposure levels are generally low in adults, averaging 0.043 mg/kg/day in Canada and 0.073 mg/kg/day in the U.S. [32], fetal, infant, and child exposure levels are much higher. In Canada, infants were found to consume up to 1.32 mg /kg/day [32], and in Europe the estimates for child BPA exposure were as high as 13 mg/kg/day [36,37]. Of particular concern regarding NDD pathophysiology, analysis of free versus conjugated BPA in human fetal samples demonstrated a reduced capacity of the fetus to metabolize BPA [38]. In this study, measurement of BPA in fetal liver samples indicated a geometric mean of 2.26 ng/g BPA/wet weight, with individual samples measuring as high as 50 ng/g (or 50 mg/kg) [38]—higher than the daily recommended exposure limitations for BPA in some countries. 

Like many other EDCs, BPA can elicit non-monotonic dose responses [39,40], which yield U-shaped dose response curves instead of linear dose response curves. BPA may also cause distinct responses depending on the developmental time point of exposure. Thus, depending on the dose administered and duration of exposure, a higher dose of BPA can elicit a milder phenotype than a lower dose [22,40,41], meaning that, when a particular dose of BPA fails to elicit a negative health impact, it cannot be assumed that all lower doses are safe. 

Consistent with the brain being a commonly reported target of EDCs [42], early BPA exposure is associated with behavioral impairment and NDDs in children [7,8,9]. Longitudinal studies that measured maternal BPA exposure levels during pregnancy and subsequently examined the children identified positive correlations between BPA and ADHD-related symptoms [43], learning deficits [44,45], externalizing behaviors [46], and anxiety and depression [47]. Cross-sectional and case-controlled studies examining concurrent BPA exposure levels found positive correlations between BPA exposure and both ASD [48,49] and ADHD [50,51]. A recent longitudinal study went a step further by examining both behavior and diffusion magnetic resonance images of children’s brains. In this study, analysis of 98 mother-child pairs revealed higher maternal urinary BPA concentrations at mid-gestation correlated with both internalizing problems and altered white matter microstructure when their children reached preschool age [52]. 

While studies examining the impact of BPA on brain structure in humans are rare, numerous studies have investigated the developmental neurotoxicity of BPA by examining the brains of animal models or cultured neurons. Here, we describe recent studies that have shed light on some of the cellular and molecular mechanisms by which BPA interferes with brain development to cause behavioral endophenotypes common to NDDs. 

## 2. Neural Stem Cell Proliferation and Differentiation Are Affected by BPA

One of the first critical cellular processes of neurodevelopment is neurogenesis, a tightly regulated process that involves the migration, proliferation, and differentiation of neural precursor cells into functional units of circuitry [53,54]. During this process, neural stem cells (NSCs) and neural progenitor cells (NPCs) transition into neurons and glia [55]. Neurogenesis begins during embryonic brain development and continues in localized areas of the adult mammalian brain, including the subventricular zone and the dentate gyrus in the hippocampus [55,56]. 

Disruptions and abnormalities in neurogenesis have been implicated in various NDDs and neuropsychiatric illnesses [57,58]. Neurogenesis can be investigated at the cellular and molecular levels by evaluating proliferation rates and expression patterns of key genes and proteins during differentiation of NSCs/NPCs. Proper proliferation and differentiation of precursor cell types relies heavily on intrinsic factors, such as mitogens, small molecules, and cell signaling pathways, but are also influenced by extrinsic factors, such as environmental chemical exposures [59,60,61,62,63,64,65,66]. Indeed, maternal exposure to BPA has been studied in various model organisms and has been shown to influence the proliferative capacity of NSCs and neuronal differentiation (Table 1) [54,67,68,69,70,71,72,73,74].

As the developing nervous system is highly complex, in vitro assays using cell-based models of neurogenesis have become a valuable tool to study NSC/NPC proliferation and differentiation [75]. Following exposure to BPA, disruptions in rates of cell proliferation and neuronal differentiation have been observed in NSCs/NPCs and neuron cultures derived from the rat hippocampus [54,71], embryonic and fetal brain [68,69], the mouse brain [72], human umbilical cord blood [70], and human brain-derived cell lines [73,74] in response to various dosing regimens of BPA. In NSCs derived from the rat hippocampus and in human brain-derived cells, BPA was found to cause a significant decrease in BrdU-positive [71] and β-III tubulin-positive cells [71,74], which are biomarkers of neuronal proliferation and differentiation, respectively. In cells derived from rat embryonic and fetal brains, BPA was also found to have adverse effects on the processes of neuronal and glial maturation [68,69]. Upon exposure to concentrations of BPA ranging from 0.05 to 100 μM, decreases in neuron and oligodendrocyte maturation were observed as was an increase in astrocyte differentiation [68]. 

BPA exposure has also been found to affect regions of the brain important for neuronal and glial homeostasis. Rats chronically exposed to 100 μg/L BPA during prenatal development and postnatal weaning were found to have significantly enlarged lateral ventricles [76]. Lateral ventricle enlargement was speculated as being caused by a BPA-induced increase in cerebrospinal fluid (CSF) pressure and circulation [76], which could directly affect developing neurons and glia given the critical role CSF plays in maintaining brain homeostasis. 

Investigators have also identified multiple molecular mechanisms by which BPA exposure disrupts the differentiation of cultured NSCs/NPCs. One such mechanism is the BPA-mediated disruption of the Wnt pathway, a signaling pathway that influences NSC/NPC development and a known contributor to the pathophysiology of NDDs and neuropsychiatric disorders [77,78]. BPA treatment was found to significantly reduce the activity of the Wnt pathway, demonstrated by altered expression of Wnt pathway genes, as well as reduced cellular β-catenin levels, reduced phosphorylation of GSK-3β (Glycogen Synthase Kinase 3 Beta), and reduced β-catenin translocation to the nucleus [54,71]. Transcription factors critical for neuronal differentiation are also impacted by BPA treatment. One study using cells derived from human umbilical cord blood showed decreased mRNA and protein levels of Sox1, Pax6, and Ngn1, and increased levels of Oct4 and Gdf3 [70]. Sox1, Pax6, and Ngn1 are actively expressed during neuronal differentiation, while expression of multipotential markers, such as Oct4 and Gdf3, is repressed [70,79]. Another analysis using human brain-derived NSCs found that concentrations of BPA as low as 1 μM resulted in a decrease in GFAP (Glial fibrillary acidic protein) and MAP2 (Microtubule associated protein 2) protein expression (markers of neuronal differentiation) as well as an increase in Nestin and Sox2 expression (markers of NSC maintenance) [73]. 

Although not as extensive as in vitro studies, there have also been studies investigating the impact of BPA on NSC/NPC proliferation and differentiation in vivo. For example, Tiwari et al. found that perinatal exposure in rats to 40 and 400 ug/kg/day caused a significant reduction in BrdU-positive NPCs in the hippocampus and subventricular zone (SVZ) of offspring [54]. Further, immunofluorescent labeling of lineage-specific markers showed BPA exposure caused a significant increase in GFAP-positive cells (a glial marker) and a concomitant decrease in nestin, b-tubulin, neuroD1, and doublecortin (DCX) expression (neuronal markers) [54]. This data suggests that early BPA exposure can increase glial differentiation and decrease neuronal differentiation, therein, disrupting the ratio of glia to neurons in the rat hippocampus. BPA was also shown to cause premature neurogenesis in the hypothalamus of zebrafish brains at concentrations as low as 0.0068 μM [67]. Another study using fruit flies demonstrated that embryonic and larval exposure to 1 mM BPA caused a reduction in the number of mitotically active cells in the larval brain [41]. 

The ability of NSCs/NPCs to proliferate and differentiate at appropriate rates is critical for neurogenesis and brain function. Both in vitro studies with cells derived from mammalian models and in vivo studies using vertebrate and invertebrate models have provided evidence that BPA exposure disrupts NSC/NPC proliferation. The delineated mechanisms involve BPA-mediated impairment of signaling pathways and gene expression critical for NSC/NPC development. Given the key role neurogenesis plays in brain development, this data demonstrates that BPA may confer a risk of NDDs by interrupting NSC/NPC development.

**Table 1:** Summary of studies on BPA-associated impacts on neural stem cell (NSC) development.

Neural Structure/ Cell Type	Organism	Phenotype	Concentration	Reference
Central brain (larval)	*Drosophila* *melanogaster*	Reduced proliferation of neuroblasts	1 mM (developmental)	Nguyen et al., 2021 [41]
Hypothalamus	*Danio rerio*	Premature neurogenesis	0.0068 μM (developmental)	Kinch et al., 2015 [67]
Neural progenitor cells	*Mus musculus*	High concentration (>100 μM) resulted in decrease in proliferation	1 nM–500 μM (in vitro)	Kim et al., 2007 [72]
Fetal neural stem cells	*Rattus* *norvegicus*	Increased cell proliferation; decreased maturation of oligodendrocytes and neurons; increased astrocyte differentiation and morphological changes; reduced arborization by astrocytes, oligodendrocytes, and neurons	0.05 μM, 0.25 μM, 10 μM, 50 μM, and 100 μM (in vitro)	Gill and Kumara., 2021 [68]
Lateral ventricles	*Rattus* *norvegicus*	Enlargement of lateral ventricles	100 μg/L; equivalent to 10 μg/kg/day (perinatal)	Santoro et al., 2021 [76]
Primary neuronal cultures from embryonic rat brains	*Rattus* *norvegicus*	Reduced maturation of neural progenitor cells (at 200 µM)	50, 100, or 200 μM (in vitro)	Cho et al., 2018 [69]
Hippocampus and lateral ventricle (in vivo); hippocampal neural stem cells (in vitro)	*Rattus* *norvegicus*	Impaired neural stem cell proliferation and differentiation (hippocampus and subventricular zone); altered expression/protein levels of neurogenic genes (hippocampus); reduced Wnt pathway activity (hippocampus)	4, 40, and 400 μg/kg/day (perinatal)	Tiwari et al., 2015 [54]
Hippocampus (in vivo) and hippocampal neural stem cells (in vitro)	*Rattus* *norvegicus*	Inhibited hippocampal-derived neural stem cell proliferation and differentiation	40 μg/kg/day (perinatal)	Agarwal et al., 2016 [71]
Neural stem cells	*Homo sapiens*	Promoted cell proliferation (0.1 and 1 µM); inhibited differentiation (1 µM); reduced GFAP and MAP2 expression (1 µM); increased expression of nestin and Sox2 (1 µM)	0.1, 1, 5, and 10 µM (in vitro)	Dong et al., 2021 [73]
Fetal brain- derived neural progenitor cells	*Homo sapiens*	Reduced neuronal differentiation (decreased β III-tubulin mRNA levels and β III-tubulin-positive cells)	10^−16^, 10^−13^, and 10^−10^ M (in vitro)	Fujiwara et al., 2018 [74]
Neural stem cells from umbilical cord blood	*Homo sapiens*	Reduced NSC proliferation and differentiation	50 and 100 μmol/L (in vitro)	Huang et al., 2019 [70]

## 3. Synapse Formation Is Disrupted by BPA

The process of synapse formation, or synaptogenesis, occurs when a neuron responds to guidance cues by extending its axon toward a target cell (a neuron, gland, or muscle cell) and forms an adhesion that enables neuronal communication across a synapse. Throughout the central and peripheral nervous systems, synaptogenesis gives rise to the specialized neural networks responsible for receiving and integrating sensory stimuli in order to actuate functional responses [80,81,82]. Thus, the input–output mechanisms of the brain that facilitate appropriate behavioral responses are dependent on successful synapse formation [80,81,82]. 

NDDs, which are often behaviorally-defined, can be caused by disruptions in synaptogenesis [80]; therefore, elucidating the impacts of BPA on synapse formation is critical because of the implications for risk of neurodevelopmental impairment. Experimentally, synapse formation can be measured at the cellular and molecular level by evaluating axon growth and guidance [41,83,84,85], dendrite length and arborization patterns [86,87,88], and the expression levels of genes and proteins critical for the synaptogenic program [89]. BPA-associated deficits in synapse formation have been examined in the fruit fly [41,85], zebrafish [83,84], rodents [87,88,89], and embryonic stem cell-derived models from humans (Table 2) [86].

Early exposure to BPA has been found to dysregulate axon growth and guidance in fruit flies and zebrafish. In the fruit fly, developmental exposure to 0.1, 1, and 2 mM BPA caused axon guidance defects in a midbrain structure called the mushroom body [41], a neural structure critical for learning and memory [90,91]. Axonal branching has also been examined at the larval neuromuscular junction (NMJ)—a relatively accessible and highly specialized synapse between the nerve terminals of motor neurons and larval body wall muscle fibers [80,92]. Analysis of the NMJ in fruit fly larvae showed that exposure to 1 mM BPA can increase axonal branching, which corresponded to transcriptomic data indicating that BPA causes the misexpression of genes involved in axogenesis and synapse development [85]. In zebrafish, exposure to 15 mM BPA decreased the length of dorsal and ventral axons extending from secondary NMJ motoneurons [84]. A separate study using zebrafish also demonstrated a BPA-mediated inhibition of motor axon growth, this time showing a dose-dependent decrease in motor axon length following exposure to 15–90 mM during embryogenesis [83]. These studies indicate that developmental exposure to BPA can have adverse effects on axon growth and guidance.

BPA-associated impacts on synapse formation have also been measured in mammalian model organisms and cell lines [87,88,89]. Similar to the reduced axon outgrowth observed in zebrafish [83,84], BPA treatment of human embryonic stem cell-derived NSCs led to a reduction in neurite length [86]. In contrast, analysis of dendritogenesis in cultured rat hippocampal neurons found treatment with 10–100 nM BPA increased the motility and density of dendritic filipodia, as well as dendrite length [87]. Studies focused on synaptic morphology have revealed BPA can also affect synaptogenesis after the presynaptic and postsynaptic membranes come into contact. Analysis of the hippocampus in male mice following perinatal BPA exposure (0.4–4 mg/kg/day) revealed altered structural characteristics of synapses, including enlarged synaptic clefts, reduced active zones, thinned postsynaptic densities, and increased synaptic curvature [88]. Cultured mouse neuroblasts (Neuro-2a/N2a cells) exhibited cell shrinkage and a reduced number of synapses upon exposure to BPA ranging from 50 μM to 200 μM [89]. BPA also caused reduced expression of Dbn (Drebin), MAP2, and Tau, and increased expression of SYP (Synaptophysin) in N2a cells [90]. Dbn and SYP regulate synaptic morphology and MAP2 and Tau are critical for maintaining stability of the neuronal cytoskeleton. Thus, the BPA-induced misexpression of these critical synaptic proteins provides a molecular explanation for its deleterious impacts on synapse formation and synaptic integrity. 

Studies using invertebrate and vertebrate models, in addition to cultured cells, provide evidence that BPA exposure can impair synapse formation by disrupting axogenesis, dendritogenesis, and synaptic structural integrity. BPA has been found to impact the expression of genes critical for synapse formation in invertebrate and mammalian systems [86,90], suggesting that BPA interferes with synaptogenesis at least in part through its dysregulation of neurodevelopmental gene expression. 

## 4. Synaptic Plasticity Is Impaired by BPA

Synaptic plasticity—the ability of neurons to modify the strength of their connections in an activity-dependent manner—is the foundation of learning and memory, adaptive social, and emotional behavior and is thought to be a common feature of many NDDs [93,94,95,96,97]. Synaptic plasticity can be measured at the cellular level by assessing dendritic spines, small protrusions along the dendritic membrane where synapses form. The density of dendritic spines changes in response to neural activity; long-term potentiation (LTP) increases spine density [98], whereas long-term depression (LTD) reduces spine density [99]. 

Chemical exposures can disrupt synaptic plasticity by altering the expression or activity of proteins important for either structural changes in dendritic spines or neuronal signaling pathways that contribute to LTP and LTD. Maternal exposure to BPA has been found to affect the synaptic plasticity of offspring in various brain regions by reducing dendritic spine density [100,101,102], altering LTP and LTD [103,104], and dysregulating the expression of molecular regulators of plasticity (Table 3) [88,101,102,103,104,105,106]. 

The impact of BPA on synaptic plasticity in the hippocampus has been extensively investigated because of the key role hippocampal plasticity plays in learning and memory [96]. Reductions in spine density have been observed in the hippocampus of non-human primates [107], rats [100,101,105,106], and mice [88,108] following prenatal or perinatal exposure to BPA at levels ranging from a very low dose (30 μg/kg/day) up to a high dose (50 mg/kg/day). Analysis of potential molecular causes of reduced hippocampal spine density suggests BPA can downregulate critical synaptic proteins, including presynaptic synapsin I [88,104], postsynaptic density protein 95 (PSD-95) [88,105,106,109], N-methyl-D-aspartate (NMDA) receptor subunits [88,105,106,110], α-amino-3-hydroxy-5-methyl-4-isoxazole propionic acid (AMPA) receptor subunits [105,106], and activity-regulated cytoskeleton-associated protein (Arc), as well as reduced PKC/ERK/CREB signaling [105]. While most of these studies exclusively used male animals, two studies that included both sexes reported different impacts in females and males (males were found to have a more robust decrease in spines) [100,105], one study found BPA-associated effects on plasticity were mediated by ER-a [105], while another study suggested plasticity phenotypes were sex-independent because BPA-associated impacts were indistinguishable in male and female brains [101]. Although there remains some debate about estrogen signaling being involved in BPA-mediated consequences in the central nervous system, all of the referenced studies found that exposure to BPA during development reduced plasticity in the hippocampus, therein, supporting the notion that maternal BPA exposure can cause learning and memory deficits in offspring. 

BPA-associated changes in synaptic plasticity have also been identified in the primary visual cortex [102], basal ganglia [104], and basolateral amygdala (BLA) [103] of rats. In the primary visual cortex, reductions in spine density were observed in response to low BPA exposure levels (1 mg/kg/day) [102]. In this case, the reduced spine density was connected to diminished expression of the proinflammatory cytokine, interleukin 1β (IL-1β) [102], which plays a role in LTP [111]. In the basal ganglia, low dose BPA (20 μg/kg/day) caused deficits in the development of LTP and LTD at the dorsolateral striatum via enhancement of dopamine receptor (D1R) activity, a disruption suggested by the authors to diminish control of motor behaviors [104]; though, it is worth noting the basal ganglia is also critical for non-motor functions, such as emotion and executive function [112]. In the BLA, low dose BPA exposure also caused increased dopaminergic signaling, which triggered elevated LTP in the cortical-BLA pathway [103], neuronal excitability thought to underpin hyperactivity and attention deficits. 

Although this review does not focus on adult exposure, numerous studies have found postnatal BPA treatment also has deleterious impacts on synaptic plasticity—typically in the form of reduced dendritic spine density—in the prefrontal cortex and hippocampus of non-human primates [113,114], rats [115,116,117,118,119], and mice [120]. The ability of BPA to consistently impair synaptic plasticity in a variety of brain structures critical for cognition and behavior in mammalian models, including non-human primates, rats, and mice, provides compelling evidence that BPA is a risk factor for NDDs in humans, given that aberrant neuroplasticity is a hallmark of these disorders. 

**Table 3:** Summary of studies that have investigated the impact of BPA on synaptic plasticity.

Brain Region	Organism	Phenotype	Exposure	Reference
Hippocampus	*Mus musculus* (males only)	Downregulated expression of PSD95 and synaptophysin; upregulated gephyrin (inhibitory); reduced excitatory to inhibitory protein ratio	50 μg/kg/day (perinatal)	Kumar and Thakur, 2017 [109]
	*Mus musculus* (males only)	Reduced spine density	40 or 400 μg/kg/day (prenatal)	Kimura et al., 2016 [108]
	*Mus musculus* (males only)	Reduced synapsin I, PSD-95, NMDA receptor subunit NR1, AMPA receptor subunit GluR1	0.04, 0.4, or 4.0 mg/kg/day (perinatal)	Xu et al., 2013 [88]
	*Mus musculus* (males only)	Downregulated NMDA receptor subunits NR1, NR2A, and 2B	50, 5, 0.5, or 0.05 mg/kg/day (perinatal)	Xu et al., 2010 [110]
	*Rattus norvegicus*	Reduced spine density in males; increased spine density in females at estrus, but reduced spine density at proestrus	30 ug/kg/day (perinatal)	Kawato et al., 2021 [100]
	*Rattus norvegicus*	Downregulated expression of *p*-NR2B, NR2B, *p*-GluA1, GluA1, PSD-95, synapsin I, PKC, *p*-ERK and *p*-CREB in males and females (greater reduction in males)	1 and 10 μg/mL, equivalent to 0.14 or 1.4 mg/kg/day (perinatal)	Wu et al., 2020 [105]
	*Rattus norvegicus*	Reduced spine density; increased mIPSC amplitude; reduced Arc (activity-regulated cytoskeleton-associated protein) expression	0.15–7.5 mg/kg/day (prenatal and postnatal, through PND 87)	Liu et al., 2016 [101]
	*Rattus norvegicus* (males only)	Reduced expressions of synaptophysin, PSD-95, spinophilin, GluR1 and NMDAR1	0.05, 0.5, 5, or 50 mg/kg/day (perinatal)	Wang et al., 2014 [106]
	*Macaca mulatta* (females only)	Reduced spine synapses in CA1, but not PFC	125 mg delivered subcutaneously to pregnant females or 50 days (resulted in mean serum level of 0.91 ± 0.13 ng/mL)	Elsworth et al., 2013 [107]
Primary visual cortex (V1)	*Rattus norvegicus* (males only)	Reduced spine density and maturity; decreased interleukin 1β (IL-1β) expression; reduced P38 phosphorylation	1 mg/kg/day (perinatal and neonatal)	Hu et al., 2020 [102]
Basal ganglia (dorsal striatum)	*Rattus norvegicus* (males only)	Caused deficits in development of LTP and LTD at dorsolateral striatum; dysregulated dopaminergic signaling (D1R and D2R)	20 μg/kg/day (perinatal and neonatal)	Zhou et al., 2009 [104]
Basolateral amygdala (BLA)	*Rattus norvegicus* (males only)	Increased neuronal excitability and facilitation of LTP induction in cortical-BLA pathway; GABAergic disinhibition; dopaminergic enhancement	2 μg/kg/day (perinatal)	Zhou et al., 2011 [103]

## 5. Behavior Is Impacted by BPA

Disruptions in neural development can have lasting effects on animal behavior. Consistent with the extensive impacts BPA elicits on the developing brain of laboratory animals, a variety of behavioral aberrations have been attributed to BPA exposure. Notably, many of the predominant BPA-associated behavioral outcomes are common endophenotypes of neurodevelopmental disorders, such as ADHD, ASD, and ID, including increased locomotor activity (hyperactivity), deficits in learning and memory, and increased anxiety-like behavior (Table 4). There are also studies indicating BPA causes contrasting phenotypes for each behavior; as with any toxicology study, these disparities may be due to differences in exposure regimen (dose, mode, and duration of exposure), genetic composition of the organism (even subtle genetic differences can alter an organism’s response to environmental toxicants [121]), or experimental design, including differences in the age at which behavioral analysis occurred. 

Despite differences in animal model and experimental design, studies examining locomotor activity have largely found developmental exposure to BPA gives rise to hyperactive progeny. At least eight separate studies using fruit flies [41,122,123], zebrafish [67,124,125], mice [126], and rats [103], found that when developing organisms were exposed to BPA, they exhibited increased locomotor activity as larvae/juveniles and/or as adults. In one study, BPA-associated hyperactivity was sex-specific and only observed in female mice [126], yet another study that solely examined locomotor activity in male rats detected hyperactive behavior [101]. Of the investigations attempting to delineate the cellular or molecular underpinnings of locomotor changes, studies using *Drosophila* and zebrafish attributed hyperactivity to BPA-mediated transcriptional changes in neurodevelopmental [85,101] and metabolic pathways [101]. Another group attributed the BPA-associated hyperactivity in rats to increased neuronal excitability in the cortical-basolateral amygdala (BLA) pathway [103]. Studies of human brains found individuals with ADHD have altered connectivity between the amygdala and prefrontal cortex, in addition to smaller amygdalae [127]. Thus, the finding that BPA impacts the cortical-BLA pathway in rats points to a potential pathophysiological mechanism underlying the association between BPA exposure and ADHD in humans [51]. In contrast to the studies reporting BPA-associated hyperactivity, three studies using *C. elegans* (in a head thrashing test) [128], zebrafish (in swimming activity tests) [84], and rats (in an open-field test) [106] found BPA exposure caused hypoactivity. 

Learning and memory involves the acquisition of information in response to environmental stimuli, which is encoded and stored in the brain for future retrieval. At least 14 studies have found early BPA exposure causes learning and memory deficits in offspring, in agreement with the many reports on BPA-associated diminishment of synaptogenesis and synaptic plasticity in the mammalian hippocampus described in preceding sections of this review. Avoidance learning and memory were found to be affected in female mice (only females were tested) [129], male mice (only males were tested) [110], and in male rats (both sexes were tested) [105]. Spatial learning and memory were disrupted by BPA in organisms ranging from zebrafish [125], mice [110], deer mice [130], and rats [101,105,106,131,132,133,134,135,136]; some studies reported sex-specific differences [130,131,135], some only tested one sex [84,132], while others found no difference on the impact in male and female rodents [101,105,125,126,133]. Object recognition learning and memory in rodents was also impaired by BPA [135,136]. In contrast, contextual fear memory was enhanced in female mice exposed to BPA [137]. In *Drosophila*, learning was found to be disrupted in males (only males were tested) in an associative learning paradigm called conditioned courtship suppression [85]; while flies do not have a hippocampus, this finding is consistent with BPA-mediated axon guidance defects of the mushroom body [41]. 

Some of the proposed underlying molecular mechanisms of BPA-mediated learning and memory impairments in rodents include modulation of PKC/ERK and BDNF/CREB signaling cascades [105,129,135], and altered expression of NMDA receptor subunits [105,106,110], AMPA receptor subunits [106], and critical pre/post-synaptic proteins [105,106], all of which can interfere with normal synaptic function. Finally, while the reasons for the disparities are unclear, some investigations found BPA had no impact on learning and memory in rodents, including two studies that reported no observed effects on spatial learning and memory [138,139], and another that found avoidance learning was intact [140]. 

Studies aimed at determining whether BPA causes anxiety have predominantly found developmental exposure leads to increased anxiety-like behaviors in offspring, often in a sex-specific manner. Three investigations using rodents of both sexes identified an increase in anxiety-like behaviors in males but not females [131,140,141]. Another study exclusively used male rodents and found similar BPA-mediated increases in anxiety-like behavior [109]. Only one study noted anxiety was increased in female rodents exposed to BPA but not males [133], and one investigation found that BPA led to a reduction in anxiety in both sexes [134]. These two studies used a Y-maze test, while all of the aforementioned studies used the open field test and/or elevated plus maze test to measure anxiety-like behavior; thus, the discrepancy may relate to the experimental modality and suggests using multiple paradigms within each study is warranted. Of the groups that identified BPA-associated anxiety-like behavior, Kumar et al. found perinatal exposure to BPA alters the ratio of excitatory to inhibitory synaptic densities [109]. Imbalances in neural excitation and inhibition have been reported in the pathophysiology of many neuropsychiatric disorders, including ASD, ADHD, schizophrenia, and epilepsy [142,143,144]. Thus, if BPA can indeed disrupt the excitation/inhibition balance, developmental exposure poses risk for numerous mental disorders. 

Several recent reviews have also discussed neurobehavioral ramifications of BPA exposure in animal models [145,146,147,148,149]. Although not reviewed here, many other behaviors have been associated with early BPA exposure, including visual perception [85,102], depression-like behavior [140,150], and social and reproductive behaviors [141,146,151]. 

**Table 4:** Summary of studies that have investigated the impact of BPA on animal behavior.

Behavior	Organism	Phenotype	Exposure	Reference
Locomotor Behavior	*Caenorhabditis* *elegans*	Reduced activity	0.01–10 mM (developmental)	Zhou et al., 2016 [128]
	*Drosophila* *melanogaster*	Increased activity	0.1–1 mM (developmental)	Musachio et al., 2021 [122]
	*Drosophila* *melanogaster*	Increased activity	0.1–1 mM (developmental)	Nguyen et al., 2021 [41]
	*Drosophila* *melanogaster*	Increased activity	0.1–1 mM (developmental)	Kaur et al., 2015 [123]
	*Danio rerio*	Increased activity (specifically in response to 0.001 μM BPA)	0.1 nM to 30 μM (developmental)	Olsvik et al., 2019 [124]
	*Danio rerio*	Increased activity	0.01, 0.1, or 1 μM (developmental)	Saili et al., 2012 [125]
	*Danio rerio*	Increased activity	0.1 or 1 μM BPA (developmental)	Kinch et al., 2015 [67]
	*Danio rerio*	Reduced activity	1, 5, or 15 μM (developmental)	Wang et al., 2013 [84]
	*Mus musculus*	Increased activity in females (not affected in males)	50 ng, 50 μg, or 50 mg BPA/kg/day (perinatal)	Anderson et al., 2013 [126]
	*Rattus norvegicus* (males only)	Increased activity	2 μg/kg/day (perinatal)	Zhou et al., 2011 [103]
	*Rattus norvegicus* (males only)	Reduced activity	0.05, 0.5, 5, or 50 mg/kg/day (perinatal)	Wang et al., 2014 [106]
Learning & Memory	*Drosophila* *melanogaster* (males only)	Impaired associative learning	1 mM (developmental)	Welch et al., 2022 [85]
	*Danio rerio*	Impaired learning	0.01, 0.1, or 1 μM (developmental)	Saili et al., 2012 [125]
	*Mus musculus*	Enhanced fear memory in females; no observed effect in males	250 ng/kg/day (perinatal)	Matsuda et al., 2013 [137]
	*Mus musculus* (females only)	Impaired memory retention	0.1–10 mg/kg/day (prenatal)	Jang et al., 2012 [129]
	*Mus musculus*	No observed effect on spatial learning and memory	20 μg/kg/day (perinatal)	Nakamura et al., 2012 [138]
	*Mus musculus* (males only)	Impaired spatial and avoidance memory	0.05–50 mg/kg/day (perinatal)	Xu et al., 2010 [110]
	*Peromyscus* *maniculatus* (deer mice)	Impaired spatial learning in males at 5 and 50 mg/kg, no observed effect in females	One or three doses of BPA at 50 μg, 5 mg, and 50 mg/kg feed weight	Jašarević et al., 2012 [130]
	*Rattus norvegicus*	Impaired spatial and recognition memory in males and females; Impaired passive avoidance memory in males	1 and 10 μg/mL, equivalent to 0.14 or 1.4 mg/kg/day (perinatal)	Wu et al., 2020 [105]
	*Rattus norvegicus* (males only)	Impaired object recognition memory	0.05, 0.5, 5, or 50 mg/kg/day (prenatal)	Wang et al., 2016 [135]
	*Rattus norvegicus*	Impaired spatial memory in both males and females	0.15–7.5 mg/kg/day (prenatal and postnatal, through PND 87)	Liu et al., 2016 [101]
	*Rattus norvegicus*	Impaired spatial recognition learning and memory in females at 2500 μg/kg/day	2.5 μg, 25 μg, and 2500 μg/kg/day (perinatal)	Johnson et al., 2016 [131]
	*Rattus norvegicus*	Altered spatial learning of females at 25 μg/kg/day (masculinization of female brain)	0, 25 μg, 250 μg, 5 mg, or 50 mg/kg/day (perinatal)	Hass et al., 2016 [136]
	*Rattus norvegicus* (males only)	Impaired working and reference memory	0.05, 0.5, 5, or 50 mg/kg/day (perinatal)	Wang et al., 2014 [106]
	*Rattus norvegicus* (males only)	Impaired spatial memory	2.5 mg/kg/day (perinatal)	Xu et al., 2014 [132]
	*Rattus norvegicus*	Impaired spatial memory in both males and females	40 ug/kg/day (perinatal)	Poimenova et al., 2010 [133]
	*Rattus norvegicus*	No effect on avoidance learning	15 μg/kg/day (prenatal)	Fujimoto et al., 2006 [140]
	*Rattus norvegicus*	Impaired working memory and object recognition memory	100 or 500 μg/kg/day (perinatal)	Tian et al., 2010 [134]
Anxiety-Like Behavior	*Mus musculus* (males only)	Increased	50 μg/kg/day (perinatal)	Kumar and Thakur, 2017 [109]
	*Mus musculus*	Increased in males, no effect in females	50 mg/kg/day (prenatal)	Cox et al., 2010 [141]
	*Peromyscus maniculatus* (deer mice)	Increased in males at 5 and 50 mg/kg, no observed effect in females	One or three doses of BPA at 50 μg, 5 mg, and 50 mg/kg feed weight	Jašarević et al., 2012 [130]
	*Rattus norvegicus*	Increased in females, no effect in males	40 ug/kg/day (perinatal)	Poimenova et al., 2010 [133]
	*Rattus norvegicus*	No observed effect	0.15, 1.5, 75, 750, and 2250 ppm (perinatal)	Stump et al., 2010 [139]
	*Rattus norvegicus*	Reduced anxiety	100 or 500 μg/kg/day (perinatal)	Tian et al., 2010 [134]
	*Rattus norvegicus*	Increased in males, no effect in females	15 μg/kg/day (prenatal)	Fujimoto et al., 2006 [140]

## 6. Conclusions

This review described the neurodevelopmental impacts—molecular, cellular, and behavioral—resulting from early exposure to BPA in organisms spanning the animal kingdom, from invertebrates to mammals. While the laboratory animals used in these studies largely served as models for understanding human impacts of BPA exposure, it should be emphasized that BPA clearly impaired the neurodevelopment of organisms throughout our environment, suggesting broad implications for the ecosystem. 

Behavioral changes caused by BPA in animal models are largely consistent with human behaviors associated with BPA exposure, including hyperactivity, learning deficits, and anxiety-like behavior. In animal models, the neurodevelopmental impacts associated with both low and high dose exposure to BPA during development are vast, beginning with interruptions in NSC development and extending to axon growth and guidance defects, impaired dendritogenesis, and altered synaptic plasticity. The findings presented in this review collectively indicate that developmental exposure to BPA should be regarded as a risk factor for NDDs in humans.

## Figures and Tables

**Table 2:** Summary of studies that have investigated the impact of BPA on synapse formation.

Neural Structure/Cell Type	Organism	Phenotype	Exposure	Reference
Mushroom body	*Drosophila* *melanogaster*	Increased axon midline crossing (axon guidance defect)	0.1 and 1 mM (developmental)	Nguyen et al., 2021 [41]
Neuromuscular junction (NMJ)	*Drosophila* *melanogaster*	Increased axonal branches	1 mM (developmental)	Welch et al., 2022 [85]
Motor neuron	*Danio rerio*	Reduced motor axon length and branching; reduced NMJ integrity	50 μM (developmental)	Morrice et al., 2018 [83]
Motor neuron	*Danio rerio*	Decreased ventral and dorsal axons from secondary motoneurons (specifically at 15 μM)	1, 5, and 15 μM (developmental)	Wang et al., 2013 [84]
Neuroblasts (Neuro-2A cell line)	*Mus musculus*	Cell shrinkage, rounding, and reduced number of synapses; decreased relative protein and mRNA expression levels of Dbn, MAP2 and Tau; increased the relative protein and mRNA expression levels of SYP	50, 100, 150, or 200 μM (in vitro)	Yin et al., 2020 [89]
Hippocampus (CA1 area)	*Mus musculus* (males only)	Inhibited synaptogenesis; altered synaptic structure	0.04, 0.4, and 4.0 mg/kg/day (perinatal)	Xu et al., 2013 [88]
Hippocampal neurons	*Rattus norvegicus*	Increased total length of dendrites; increased motility and density of dendritic filipodia	1, 10, and 100 nM (in vitro)	Xu et al., 2014 [87]
Embryonic stem cell-derived neural stem cells	*Homo sapiens*	Decreased neurite outgrowth	1, 10, and 100 nM (in vitro)	Liang et al., 2020 [86]

## Data Availability

Not applicable.
